# Bromination of hydrocarbons with CBr_4_, initiated by light-emitting diode irradiation

**DOI:** 10.3762/bjoc.9.190

**Published:** 2013-08-14

**Authors:** Yuta Nishina, Bunsho Ohtani, Kotaro Kikushima

**Affiliations:** 1 Research Core for Interdisciplinary Science, Okayama University, Tsushimanaka, Kita-ku, Okayama 700-8530, Japan; 2 Catalysis Research Center, Hokkaido University, Sapporo 001-0021, Japan

**Keywords:** bromination, free radical, hydrocarbon, light-emitting diode, photo irradiation

## Abstract

The bromination of hydrocarbons with CBr_4_ as a bromine source, induced by light-emitting diode (LED) irradiation, has been developed. Monobromides were synthesized with high efficiency without the need for any additives, catalysts, heating, or inert conditions. Action and absorption spectra suggest that CBr_4_ absorbs light to give active species for the bromination. The generation of CHBr_3_ was confirmed by NMR spectroscopy and GC–MS spectrometry analysis, indicating that the present bromination involves the homolytic cleavage of a C–Br bond in CBr_4_ followed by radical abstraction of a hydrogen atom from a hydrocarbon.

## Introduction

Bromination reactions of organic compounds are fundamental reactions for providing a wide variety of organic precursors for industrial materials [1–8]. Generally, the bromination of saturated hydrocarbons proceeds through radical abstraction of hydrogen atoms and trapping with bromide, whereas the bromination reactions of aromatic and unsaturated hydrocarbons are induced by electrophilic addition of bromine and/or a cationic bromide. Combinations of *N*-bromosuccinimide (NBS) with azobisisobutyronitrile or benzoyl peroxide as radical initiators are typical conditions for Wohl–Ziegler bromination [9–12] and are widely used for the bromination of benzylic and allylic positions, despite the need for heating and the generation of equimolar amounts of waste. To avoid these drawbacks, several efforts have been focused on benzylic bromination using Br_2_ or bromide salts as highly efficient bromine sources [13–17]. However, the direct bromination of non-activated C–H bonds is still a challenging task. Although Br_2_ [13], CBr_4_ [18–20], R_4_NBr [21–22] and LiBr [23] have been reported to serve as bromine sources for the bromination of saturated hydrocarbons, these reactions exhibit low selectivity or reactivity. Efficient bromination using Br_2_ as a bromine source combined with a stoichiometric base [24], an excess of MnO_2_ [25], or a catalytic amount of Li_2_MnO_3_ [26] has been reported to give high reactivity and selectivity. The combination of CBr_4_ with a copper catalyst at high temperature also achieves effective bromination of hydrocarbons [27].

We have focused on CBr_4_, which is solid and easy to handle, as a bromine source. CBr_4_ has been used in organic synthesis to give useful bromide-containing precursors. For instance, alkyl alcohols can be converted to alkyl bromides in the presence of CBr_4_ and triphenylphosphine; this is known as the Appel reaction [28]. This combination can also be used to transform aldehydes into dibromoalkenes, which are useful precursors for the Corey–Fuchs reaction [29], to obtain terminal alkynes. Although CBr_4_ has been used for various bromination reactions including radical brominations, these reactions need further additives to proceed. Here, we disclose the efficient bromination of saturated hydrocarbons, using CBr_4_ as a bromine source without any additives, through radical reactions induced by irradiation with light from commonly used light-emitting diodes (LEDs) [30]. In this reaction, additives, catalysts, heating, and inert reaction conditions are all unnecessary.

## Results and Discussion

First, the bromination of cyclohexane under LED irradiation was investigated using 1.0 mL of cyclohexane with 0.20 mmol CBr_4_ (Table 1). The desired monobrominated product was obtained in 77% yield, based on CBr_4_, after 2 h, and no dibromide was observed (Table 1, entry 1). It was found that the yield of cyclohexyl bromide exceeded 100% after 3 h (Table 1, entry 2). When the mixture was irradiated for 4 h, the product yield reached 148% and had almost peaked (Table 1, entry 3). Further improvements were not observed, even after 24 h (Table 1, entry 5). These results indicate that during the reaction one or more bromine atoms originated from one CBr_4_. It is considered that CHBr_3_ generated through radical abstraction of a hydrogen atom by a tribromomethyl radical served as a bromine source. To test this hypothesis the reaction was repeated with CHBr_3_ instead of CBr_4_ and the product was obtained in a low yield (Table 1, entry 6), whereas the reaction with CH_2_Br_2_ produced no bromination product at all under these conditions (Table 1, entry 7). Other bromination reagents such as NBS also gave the desired product in moderate yield (Table 1, entry 8). In the case of tetrabutylammonium bromide, no brominated product was obtained (Table 1, entry 9), showing that the present reaction was a radical reaction. Based on the assumption that the initial formation of bromine radicals would be important, addition of catalytic amounts of CBr_4_ along with various bromination sources was examined (Table 1, entries 10–12). The combination of catalytic CBr_4_ with CHBr_3_ or CH_2_Br_2_ resulted in slight improvements in the yields (Table 1, entries 10 and 11), showing these bromides also could serve as bromination sources in the presence of the radical species. The combination of CBr_4_ with NBS gave the desired product in a moderate yield (Table 1, entry 12). On the other hand, the reaction was inhibited by the addition of water (Table 1, entry 13) and performing the reaction under inert argon atmosphere led to a decreased yield of 87% (Table 1, entry 14).

**Table 1:** Bromination of cyclohexane using CBr_4_ under LED irradiation.^a^

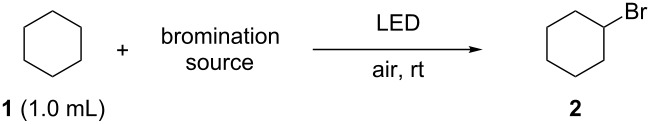			

entry	bromination source (mmol)	time (h)	yield (%)^b^

1	CBr_4_ (0.20)	2	77
2	CBr_4_ (0.20)	3	108
3	CBr_4_ (0.20)	4	148
4	CBr_4_ (0.20)	5	148
5	CBr_4_ (0.20)	24	150
6	CHBr_3_ (0.20)	24	27
7	CH_2_Br_2_ (0.20)	24	0
8	NBS (0.20)	24	31
9	Bu_4_NBr (0.20)	24	0
10	CBr_4_ (0.02)/CHBr_3_ (0.20)	24	39
11	CBr_4_ (0.02)/CH_2_Br_2_ (0.20)	24	16
12	CBr_4_ (0.02)/NBS (0.20)	24	79
13^c^	CBr_4_ (0.20)	24	0
14^d^	CBr_4_ (0.20)	24	87

^a^Conditions: 1.0 mL of cyclohexane, bromination sources, under LED irradiation, rt. ^b^Yields were determined by GC analysis based on the mole of CBr_4_. ^c^In the presence of 0.10 mL water. ^d^Under Ar.

Based on the above experiments, the bromination of other substrates was examined with CBr_4_ under LED irradiation. Cyclooctane underwent bromination under the optimized conditions to furnish the monobromide in 178% yield, based on CBr_4_, without contamination by dibromide (Table 2, entry 1). The bromination of *n*-hexane produced three bromides: 1-bromohexane (14%), 2-bromohexane (84%), and 3-bromohexane (41%) (Table 2, entry 2). On the other hand, no bromination of toluene occurred under LED irradiation. In this case, light would be absorbed by the aromatic ring of toluene, suppressing the activation of CBr_4_. Using sunlight in place of LED light, however, resulted in the bromination of the benzylic position to give benzyl bromide in 140% yield (Table 2, entry 3).

**Table 2:** Bromination of other substrates using CBr_4_ under LED irradiation.^a^

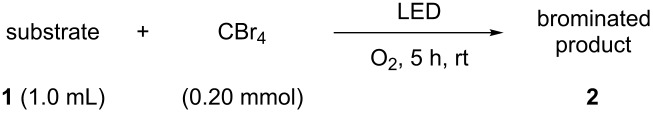			

entry	substrate	product	yield (%)

1	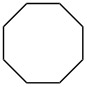	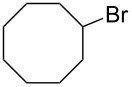	178

2	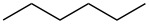	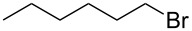	14
		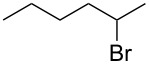	84
		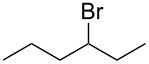	41

3	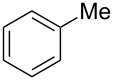	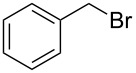	140^b^

^a^Conditions: 1.0 mL of substrate, 0.20 mmol of CBr_4_, under LED irradiation. Yields were determined by GC using dodecane as an internal standard. ^b^Under sunlight irradiation.

**Figure 1:**
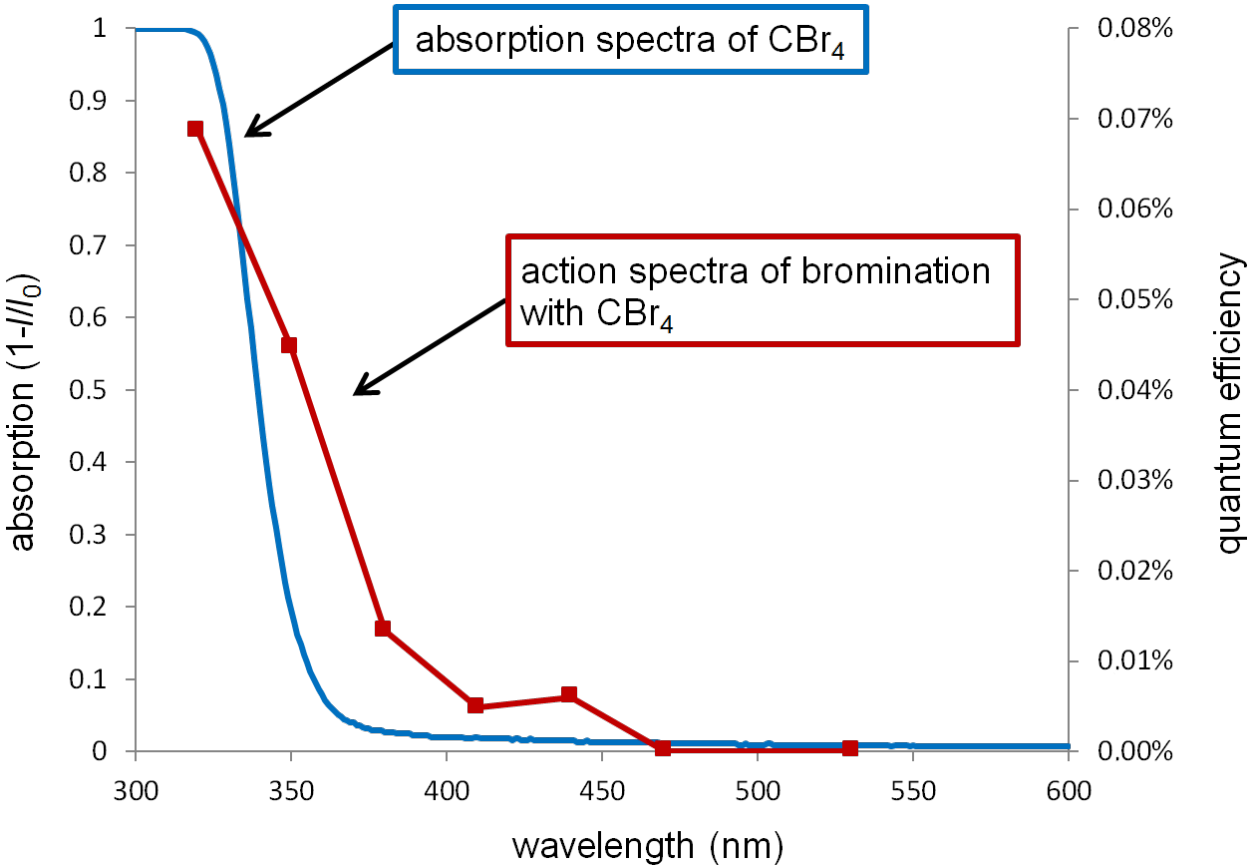
Action spectrum of bromination with CBr_4_, induced by LED irradiation (red line), and absorption spectrum of CBr_4_ (blue line).

To investigate the wavelength dependency of the present reaction, the action spectrum of the bromination of cyclohexane in the presence of CBr_4_ was obtained by plotting the apparent quantum efficiency against wavelength (Figure 1, red line) [31]. It was found that the present reaction was promoted by irradiation with ultraviolet (UV) light and deactivated under visible-light (>475 nm) irradiation. CBr_4_ shows strong absorption in the UV region (Figure 1, blue line), and this overlaps with the above-mentioned action spectrum. The activation of CBr_4_ is therefore considered to be induced by photo-irradiation, initiating the reaction. Although other light sources could also activate CBr_4_, we adopted LED light due to safety, mildness, and availability. We have confirmed that fluorescent room light could also promote the reaction.

**Scheme 1:**
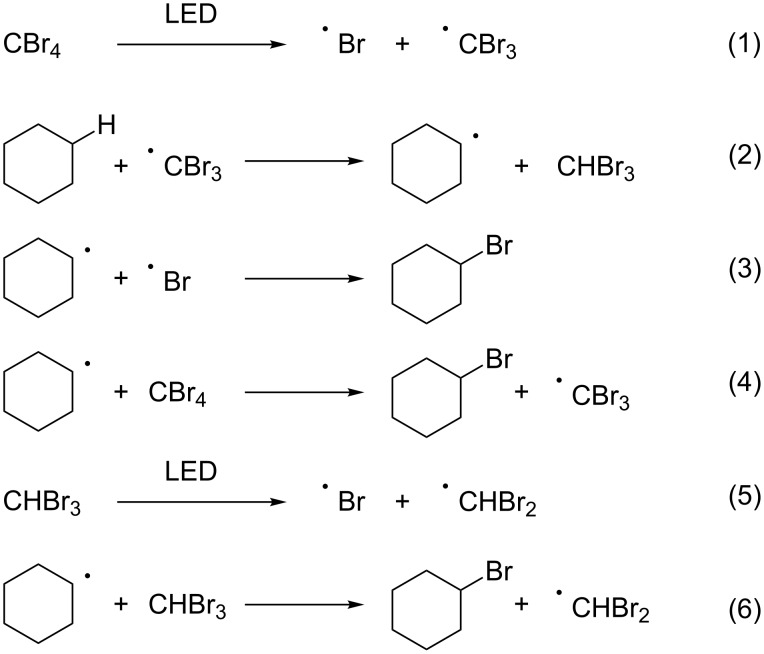
Plausible mechanism for bromination of cyclohexane with CBr_4_ induced by LED irradiation.

A plausible mechanism for the present bromination is illustrated in Scheme 1. First, photo-irradiation generates a bromine radical and a CBr_3_ radical (Scheme 1, reaction 1), which abstracts a hydrogen atom from the substrate to form CHBr_3_ (Scheme 1, reaction 2). Finally, the radical species derived from the substrate reacts with the bromine radical or CBr_4_ to afford the brominated product (Scheme 1, reactions 3 and 4). Additionally, the in situ generated CHBr_3_ releases a bromine radical upon LED irradiation, thus serving as a bromine source (Scheme 1, reaction 5). Alternatively the radical species derived from the substrate abstracts a bromine atom from CHBr_3_ (Scheme 1, reaction 6).

**Figure 2:**
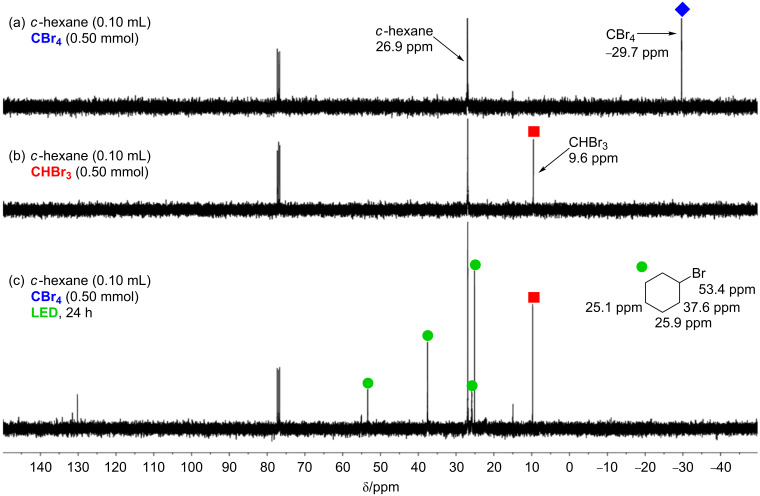
^13^C NMR monitoring of reaction mixtures: (a) 0.50 mmol of CBr_4_, 0.10 mL of cyclohexane, and 0.40 mL of CDCl_3_, (b) 0.5 mmol of CHBr_3_, 0.10 mL of cyclohexane, and 0.40 mL of CDCl_3_, (c) 0.50 mmol of CBr_4_ and 0.10 mL of cyclohexane, LED irradiation for 24 h, and then addition of 0.40 mL of CDCl_3_.

To examine the above hypothesis, the bromination of cyclohexane was monitored using ^13^C NMR spectroscopy (Figure 2). CBr_4_ (0.50 mmol) dissolved in cyclohexane (0.10 mL) and CDCl_3_ (0.40 mL) was observed at −29.7 ppm (Figure 2a). After stirring a reaction mixture of CBr_4_ (0.50 mmol) and cyclohexane (0.10 mL) under LED irradiation for 24 h, peaks assigned to bromocyclohexane (53.4, 37.6, 25.9, and 25.1 ppm) and another strong peak at 9.6 ppm appeared (Figure 2c). The latter peak was found to be consistent with the peak of CHBr_3_ (0.50 mmol) dissolved in cyclohexane (0.10 mL) and CDCl_3_ (0.40 mL) (Figure 2b). Additionally, the generation of CHBr_3_ in the present bromination was confirmed by ^1^H NMR spectroscopy and GC–MS spectrometry. These results support the reaction pathway described above, although the chemical species after the second bromination was not assigned at this point.

## Conclusion

In conclusion, we have developed a method for the hydrocarbon bromination induced by LED irradiation using CBr_4_ as a bromine source. The present reaction system did not require any additives, catalysts, heating, or inert conditions, and is therefore an extremely simple procedure. An action spectrum and NMR measurements showed that the LED irradiation activates CBr_4_ to generate bromine radicals, which initiate the bromination reaction. Further elucidation of the detailed mechanism and the use of LED irradiation in other reaction systems are under investigation in our laboratory.

## Experimental

### General information

All commercially available compounds were purchased and used as received. Cyclohexane, cyclooctane, *n*-hexane, and toluene were purchased from Wako Pure Chemical Industries and used as received. ^1^H (400 MHz) and ^13^C (100 MHz) NMR spectra were recorded using a JEOL JNM-LA400 spectrometer. Proton chemical shifts are reported relative to residual solvent peak of CDCl_3_ at δ 7.26 ppm. Carbon chemical shifts are reported relative to CDCl_3_ at δ 77.00 ppm. Gas chromatographic analysis was conducted with Shimadzu GC-2014 equipped with FID detector. The chemical yields were determined using dodecane as an internal standard. The NMR data of all brominated products match those reported.

### General procedure for the bromination induced by LED irradiation

A reaction tube was charged with CBr_4_ (66.33 mg, 0.20 mmol) and a hydrocarbon (1.0 mL). The reaction mixture was stirred under white LED (7 W) irradiation. To this was added dodecane (45.2 μL, 0.20 mmol) and the yield was determined by GC analysis with dodecane as an internal standard.
